# False Elevation of Troponin in a Case of Multiple Myeloma

**DOI:** 10.7759/cureus.34186

**Published:** 2023-01-25

**Authors:** Omer Bajwa, Gashaw Hassen, Jacob Fishbein, Unamba Uchenna, Hussam Ammar

**Affiliations:** 1 Internal Medicine, University of Maryland Capital Region Medical Center, Maryland, USA

**Keywords:** macrotroponin, false high troponin, troponin, clinical reasoning, myeloma

## Abstract

Falsely elevated troponin has been reported in the literature. The authors present a case of a 51-year-old man who was admitted with nausea, vomiting, and chest discomfort. He was found to have elevated troponin with no electrocardiographic changes. He has normal coronaries on angiogram and normal echocardiogram. A diagnostic time-out and second look at the laboratory values captured abnormalities that triggered a workup that ruled in a multiple myeloma diagnosis. We suspected falsely elevated troponin levels secondary to macrotroponin, a complex of elevated immunoglobulin levels and troponin, which has been rarely reported to cause elevated troponin levels in patients with multiple myeloma.

## Introduction

Elevated troponin is a diagnostic marker of acute coronary syndrome. It is also a red flag and a marker of increased mortality and morbidity in almost every condition associated with elevated troponin levels, such as sepsis, pulmonary embolism, tachycardia, anemia, stroke, and congestive heart failure [1]. Heterophil antibodies and autoantibodies can rarely interfere with high-sensitivity troponin assay causing falsely elevated troponin and myocardial infarction misdiagnosis [2-4].

## Case presentation

**Figure 1:**
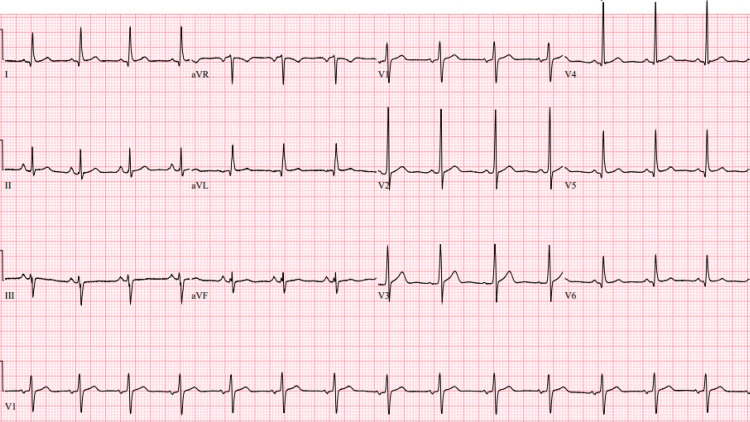
Electrocardiogram; sinus rhythm with no significant ST-T changes

**Figure 2:**
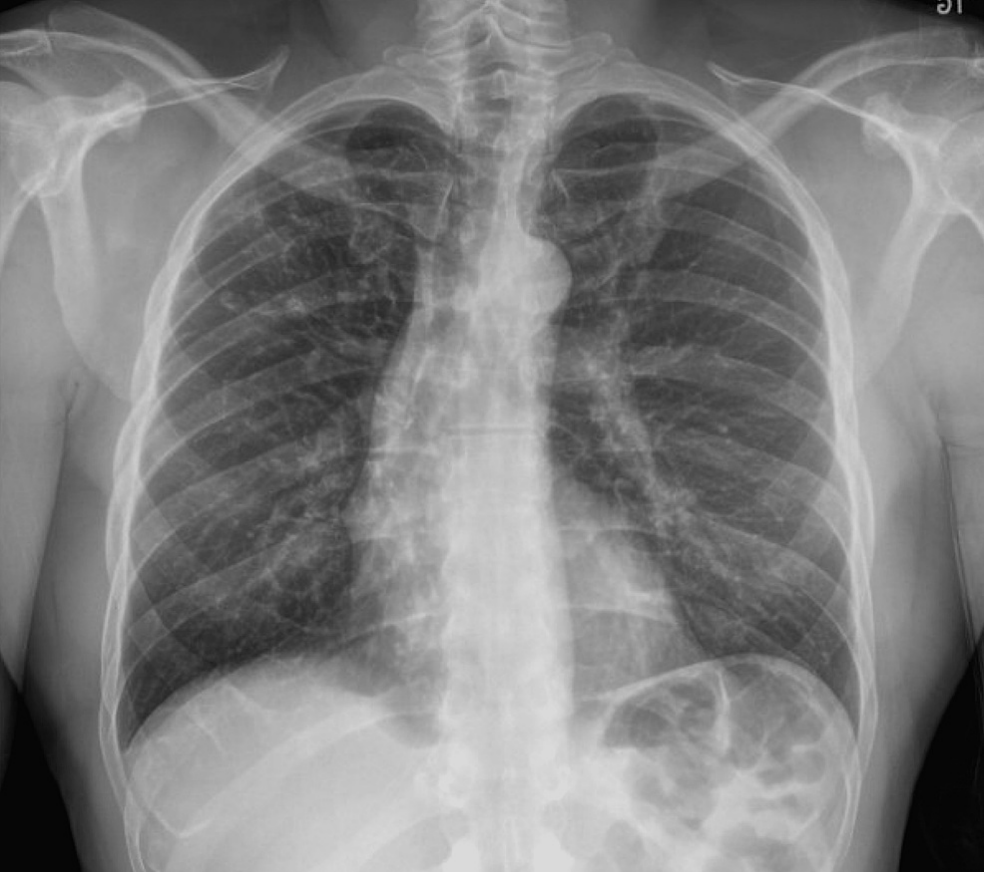
Unremarkable Chest X-ray

A 51-year-old man with no significant past medical history presented to the emergency department with acute chest and back pain. He experienced nausea and vomiting two days before this presentation; he thought his symptoms were related to eating noodles, and he also experienced subjective fever and chills. On admission, he had normal vital signs: blood pressure was 114/81 mm Hg, pulse rate was 91 per minute, temperature 37.2 C, and respiratory rate was 24 per minute. His physical exam was unremarkable. He had a normal electrocardiogram and chest radiograph (Figures 1, 2).

Admission laboratory values are listed in Table 1.

**Table 1:** Admission laboratory values

Variable	Reference range	Admission value
Troponin (ng/ml)	0.01-0.029	1.02
Hemoglobin (g/dl)	13.5-17.5	10.3
White blood cell count (per μl)	4000-11,000	6100
Platelet count (per μl)	150,000-450,000	133,000
Sodium (mmol/l)	136-145	130
Potassium (mmol/l)	3.5-5.1	3.5
Calcium (mg/dl)	8.6-10.2	8.5
Creatinine (mg/dl)	1.2	0.7-1.2
Sedimentation level (mm/h)	0-15	>105

Urine analysis was positive for 2+ protein. He had unremarkable chest radiography, and a computed pulmonary angiogram did not reveal a pulmonary embolism. Acute coronary syndrome: non-elevated ST-segment elevation myocardial infarction was the initial working diagnosis. The patient was treated with aspirin, intravenous heparin infusion, and metoprolol, and he was taken for an urgent coronary angiogram that revealed normal coronaries. Echocardiogram was unremarkable: normal Ejection fraction, ventricles, and atria were normal in dimension, there was no ventricular hypertrophy, and he has a normal valvular function.

The medicine team started to have a second look at the case. The patient did not experience any more episodes of chest pain since admission, he remained afebrile, and his nausea and vomiting resolved. The troponin plateaued but remained elevated. A total serum protein was elevated at 11.7 gm/dl. The combination of high serum proteins, anemia, and thrombocytopenia fits the illness script of myeloma. A serum protein electrophoresis revealed a Monoclonal protein of 4.7 gram/dl, and Immunoglobulin G (IgG) was elevated at 4.7 mg/dl. 24-hour urine protein was 3.4 grams. Urine immunofixation electrophoresis was positive for kappa-free light chains. Magnetic resonance imaging revealed heterogenous signals throughout the marrow with no lytic lesion. The medicine team made the diagnosis of IgG myeloma. The patient followed up with an outside hematologist and did not come back to our clinics. 

## Discussion

The treating physicians were initially concerned about acute coronary syndrome because of elevated troponin in a healthy 51-year-old man who presented with nausea, vomiting, chest, and backache. Acute coronary syndrome is common, can present without chest pain as the presenting complaint, can have a normal electrocardiogram as in this case, and missing its diagnosis can have life-threatening consequences [5]. At that point in time, the diagnosis should not be missed and should be either decisively ruled in or ruled out. This reasoning was mostly system 1 thinking decision: fast, intuitive, pattern recognition based. Cognitive psychologists hypothesized that our thinking process comes through 2 systems; system 1 is: fast, effortless, intuitive, generates complex patterns of ideas, and is susceptible to biases, and system 2: is slow, effortful, analytic, and deliberate [6,7]. A few subtle findings were overlooked in the context of adrenaline surge and concern about a life-threatening diagnosis: anemia, thrombocytopenia, high total proteins, and proteinuria. This is also typical of system 1 thinking. It lacks the analytical deductive characteristics of system 2 thinking [6,7]. The patient was admitted to the cardiovascular unit, and the coronary angiogram revealed normal coronaries; the patient's symptoms resolved, and the echocardiogram was largely unremarkable. Once the dust settled and the adrenaline surge calmed down, system 2 kicked in and raised the expected question: Why does this previously healthy man have elevated troponin, a laboratory finding associated with increased mortality in every clinical context? The treating physicians took a diagnostic time out and tried to tie the different findings in the case together. We were careful not to fall into the traps of premature closure and call the elevated troponin secondary to demand ischemia as the patient wasn't tachycardic and had normal vitals throughout his admission; his electrocardiogram and echocardiogram images also didn't suggest a diagnosis of myocarditis [1]. About 75% of patients with myeloma have anemia, 30% have hypercalcemia, 70% have bone disease, and 25% have renal impairment. Symptomatic myeloma requires the presence of monoclonal plasma cells > 10% of bone marrow cells, elevated monoclonal protein in the serum or urine, and evidence of myeloma-related organ or tissue damage. Asymptomatic myeloma requires the presence of monoclonal protein >/= 3 gm/dl or monoclonal plasma cells in marrow >/=10% and the absence of myeloma-related organ damage [8,9]. The serum and urine protein electrophoresis and immunofixation ruled in a diagnosis of IgG myeloma. The magnetic resonance imaging of the spine did reveal an abnormal bone marrow signal but without lytic lesions. Multiple myeloma was a unifying diagnosis that could easily explain the hematological, biochemical, and radiological findings. The case sounds like perfect diagnostic parsimony, except the physician could not explain the elevated troponin and gastroenteritis-like presentation [8,9]. Gastroenteritis was a self-limiting illness. It was likely a red herring that just happened and uncovered a serious diagnosis that was not symptomatic. A quick literature review revealed that cardiac amyloidosis has been described in myeloma and can be associated with high troponin; the unremarkable images of the echocardiogram and electrocardiogram argue against this diagnosis.

Few reports documented falsely elevated high-sensitivity troponin levels secondary to interference attributed to heterophils antibodies, autoantibodies, and macrotroponin. Domanski et al. reported a case of a 62-year-old with persistently elevated troponin; he had extensive cardiac testing, including an angiogram, that was unrevealing, like our case [2]. Macrotroponin is a complex of immunoglobulin and cardiac troponin rarely reported in myeloma cases, COVID vaccination, and COVID infection and can falsely elevate troponin levels [2-4]. Adding polyethylene glycol to the patient's serum precipitates immunoglobulins, frees troponin molecules, and normalizes the troponin value [2-4]. This process wasn't available in our laboratory. 

## Conclusions

The diagnosis of myeloma, in this case, was made within 48 hours of admission. The diagnostic strategy that the team used, explicitly taking a diagnostic time out, avoiding premature closure, and employing system 2 in the decision-making process, made a new diagnosis of multiple myeloma and suspected a falsely elevated troponin level.
